# A Nomogram for Predicting Multiple Metastases in Metastatic Colorectal Cancer Patients: A Large Population-Based Study

**DOI:** 10.3389/fonc.2021.633995

**Published:** 2021-05-13

**Authors:** Yuhang Ge, Renshen Xiang, Jun Ren, Wei Song, Wei Lu, Tao Fu

**Affiliations:** Department of Gastrointestinal Surgery II, Renmin Hospital of Wuhan University, Wuhan, China

**Keywords:** nomogram, multiple metastasis, colorectal cancer, risk factors, surveillance epidemiology and end results

## Abstract

**Objectives:**

The present study aims to discover the risk factors of multiple metastases and develop a functional nomogram to forecast multiple metastases in metastatic colorectal cancer (mCRC) patients.

**Methods:**

mCRC cases were retrospectively collected from the Surveillance, Epidemiology, and End Results (SEER) database between 2010 and 2016. Survival times between multiple metastases and single metastasis were compared using Kaplan–Meier analysis and log-rank tests. Risk factors for multiple metastases were determined by univariate and multivariate logistic regression analyses, and a nomogram was developed to forecast the probability of multiple metastases in mCRC patients. We assessed the nomogram performance in terms of discrimination and calibration, including concordance index (C-index), area under the curve (AUC), and decision curve analysis (DCA). Bootstrap resampling was used as an internal verification method, and at the same time we select external data from Renmin Hospital of Wuhan University as independent validation sets.

**Results:**

A total of 5,302 cases were included in this study as training group, while 120 cases were as validation group. The patients with single metastasis and multiple metastases were 3,531 and 1,771, respectively. The median overall survival (OS) and cancer-specific survival (CSS) for patients with multiple metastases or single metastasis were 19 *vs.* 31 months, and 20 *vs.* 33 months, respectively. Based on the univariate and multivariate analyses, clinicopathological characteristics were associated with number of metastasis and were used to establish nomograms to predict the risk of multiple metastases. The C-indexes and AUC for the forecast of multiple metastases were 0.715 (95% confidence interval (CI), 0.707–0.723), which showed the nomogram had good discrimination and calibration curves of the nomogram showed no significant bias from the reference line, indicating a good degree of calibration. In the validation group, the AUC was 0.734 (95% CI, 0.653–0.834), and calibration curve also showed no significant bias, indicating the favorable effects of our nomogram.

**Conclusions:**

We developed a new nomogram to predict the risk of multiple metastases. The nomogram shows the good prediction effect and can provide assistance for clinical diagnosis and treatment.

## Introduction

Colorectal cancer (CRC) is the third most commonly diagnosed malignancy and the second leading cause of cancer death, which almost cause 900,000 deaths annually (1, 2). CRC is largely an asymptomatic disease until it reaches an advanced stage; therefore, the majority of CRC patients are diagnosed at advanced stage. Advanced colorectal cancer often metastasizes through the bloodstream, lymphatic system, and intraperitoneal route, which is an important reason for poor prognosis in CRC patients (1, 2). Despite the extremely poor prognosis of mCRC, advances in epidemiological studies of mCRC have been limited.

The most common sites of metastasis are the lymph nodes, liver, and lungs in CRC. Occasionally, some special distant sites such as the bone, ovary, peritoneum, and brain may be involved. Although it is known that CRC patients with distant metastasis have a poor prognosis, there is a lack of further stratified analysis of the prognosis of patients with distant metastasis, such as exploring the difference between patients with single metastasis and multiple metastases to clarify the causality and prognostic value. Understanding the patterns of metastasis, especially multiple metastases in CRC, is vitally crucial to improving diagnosis, treatment, and health education for patients.

The current tumor-node-metastasis (TNM) staging system only evaluated whether a patient has metastasis, but it cannot predict whether the patient will have metastasis. In order to make up for the deficiency of the current TNM staging system, some related biomarkers have been explored, studied, and applied in clinical practice. For example, Mismatch repair (MMR) status or microsatellite instability (MSI) has been commonly recommended as the most used and significant molecular marker in clinical management of CRC patients (3, 4). In addition, the expression status of various genes, such as Serine/threonine-protein kinase B-Raf (BRAF) and V-Ki-ras2 Kirsten rat sarcoma viral oncogene homolog (KRAS), were also found to be closely associated with the metastasis of CRC patients (5). However, genetic testing methods have some limitations, such as only suitable for certain groups of patients and bring about certain economic burden to patients. As a result, a statistical model tool, called a nomogram, which comprehensively incorporates the effects of diverse clinical pathological factors, has become a supplement to the above method. Many nomogram scoring systems related to the CRC had been reported recently. For instance, Li et al. also proposed a nomogram, which combined clinical risk factors with radiological characteristics for the prediction of lymph node metastasis in CRC patients (6). Sun et al. built a nomogram associated preoperative plasma fibrinogen with neutrophil-to-lymphocyte ratio to predict the relapse in rectal cancer patients (7). Wang et al. constructed a competitive risk nomogram to predict the specific risk of death in elderly colorectal cancer patients (8). However, these studies lack a hierarchical analysis of metastasis, especially multiple metastases. Although some studies attempted to develop nomograms to predict metastasis, these nomograms only predicted specific metastasis site such as the liver, lung, bone, lacking a hierarchical analysis of metastasis status to distinguish single metastasis and multiple metastases for CRC (9–12). Therefore, there was almost no research that investigates the potential risk factors of multiple metastases and developed a nomogram to predict the risk of multiple metastases. The main reason why such studies are rarely carried out is the relatively limited data on patients with multiple metastases.

The primary objective of this study was to investigate the potential risk factors of multiple metastases and develop a functional nomogram to predict multiple metastases in mCRC patients by using the SEER database. Furthermore, we also selected patients from Renmin Hospital of Wuhan University as an independent external verification cohort to verify the external applicability of the nomogram.

## Materials and Methods

### Data Source and Study Design

We designed a retrospective study in a large population of mCRC patients from the SEER database. The SEER program of the United States National Cancer Institute is an authoritative source which collects patient demographic information, cancer diagnostic information, and outcomes from 18 population-based cancer registries that cover approximately 28% of the U.S. population.

**Figure 1:**
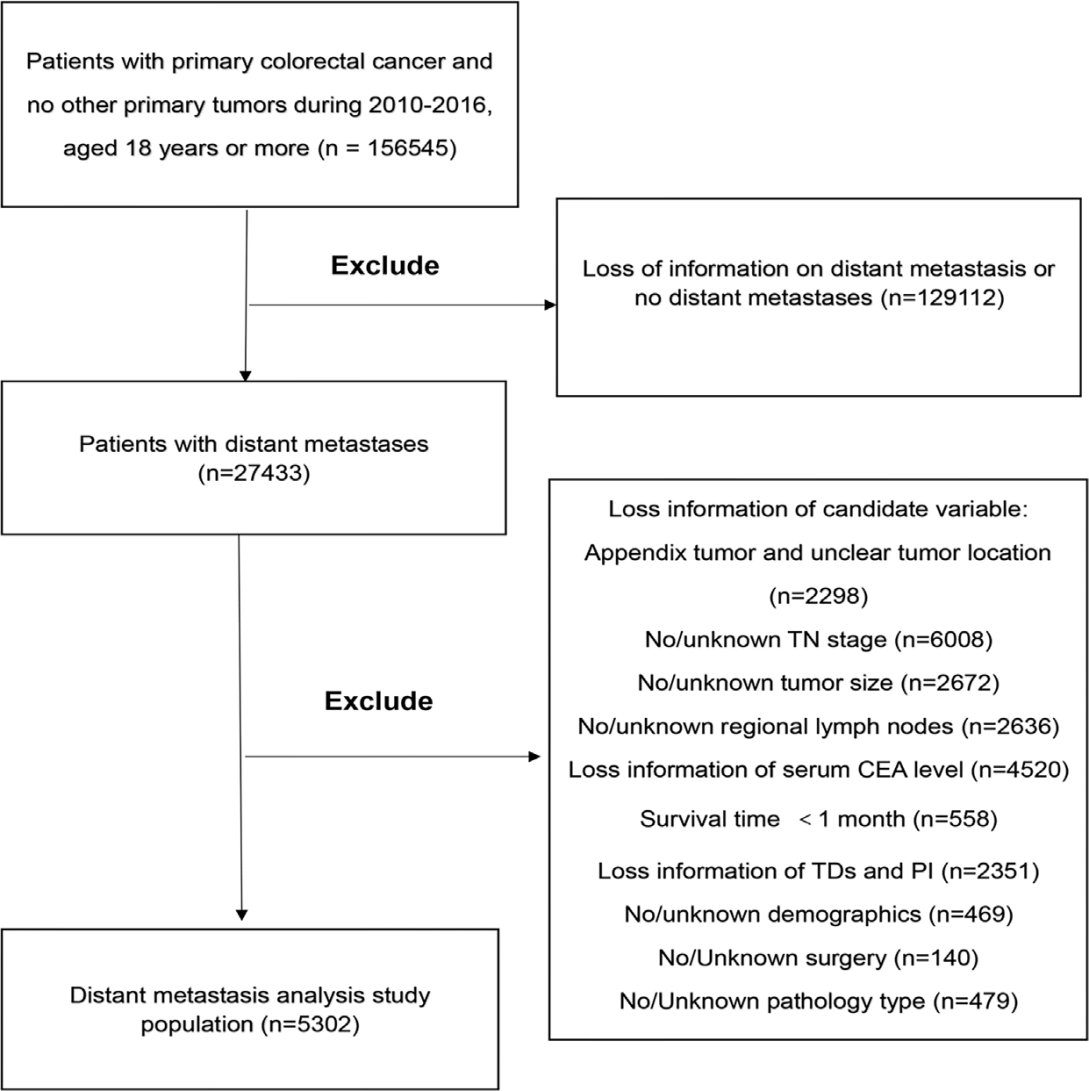
The study flow diagram of the selection process. CEA, carcinoembryonic antigen; TDs, Tumor Deposits; PI, Perineural Invasion.

We identified an open cohort in which cases are diagnosed with CRC between 2010 and 2016 employing the SEER-Stat software (SEER*Stat 8.3.6.1, http://seer.cancer.gov/seerstat/software/). In this study, the inclusion criteria were: 1) Patients diagnosed with CRC were older than 18 years old between 2010 and 2016; 2) When the patient was diagnosed, there was only one primary tumor and no multiple cancers; 3) The patient’s confirmed evidence was confirmed by the pathologist under the microscope; 4) There is clear information about distant metastasis; 5) Patients with active follow-up for at least 1 month. The exclusion criteria were: 1) Patients with incomplete information about our concerned information, including age, sex, race, marital status, insurance status, T stage, N stage, pathological grade, histological type, tumor size, tumor location, lymph node surgery scope, serum carcinoembryonic antigen (CEA) level, tumor deposits (TDs), perineural invasion (PIN), or regional node examination information; 2) Patients were diagnosed as appendix tumors; 3) Patients’ surgery information was unclear or patients without surgery. A complete flow chart describing the selection process is shown on **Figure 1**. After submitting a request to the SEER database project and obtaining permission, data freely downloaded from the SEER database did not require patients’ informed consent. In our research, a total of 156,545 cases were obtained from the SEER database. Based on the criteria described above, a total of 5,302 cases were included in the research

To further validate our nomogram, patients diagnosed as mCRC from Renmin Hospital of Wuhan University between 2017 and 2020 were included as external validation set. The validation group included 120 mCRC patients who were recruited according to the same criteria.

### Outcomes

Metastasis is characterized by the spread of cancer cells from the primary organ to other organs or tissues through the bloodstream, lymphatic system, or intraperitoneal planting (2). Outcome variable was metastatic state, which was defined as single and multiple metastases. Single metastasis included one of the liver, lung, distant lymph node, peritoneum, bone, brain, or other organs; multiple metastases contained at least two of the above metastases. In the logistic regression model, the occurrence of multiple metastases was considered an outcome event. In the SEER database, CS Mets DX project code in the Collaborative Stage (CS) project can identify single and multiple metastases. Although CS Mets DX project code was not provided in 2016, more specific metastatic sites were provided, including the liver, lung, brain, bone, distant lymph nodes, and other organs, which could still differentiate single and multiple metastases.

### Predictor Variables

We extracted data for demographic factors and clinicopathological parameters. Demographic factors included age, race, sex, marital status, and insurance status. Age was divided into two parts: below 50 years old and at least 50 years old. Race was classified into white, the black, and others (containing American Indian, Asian, and Pacific Islander). Marital status was categorized into married, never married, and others (including divorced, separated, widowed, and unmarried or domestic partner). Clinicopathological parameters included serum CEA level, tumor location, pathological grade, histological type, T stage, N stage, LODDS, tumor size, PIN, and TDs. Serum CEA level was classified into negative and positive. Tumor location was divided into proximal colon cancer, distal colon cancer, and rectum cancer. The definition of proximal colon cancer and distal colon cancer lesions were consistent with a previous study: proximal colon cancer was defined as location of the tumor, including the cecum, ascending colon, hepatic flexure, and proximal transverse colon, while distal colon included the distal transverse colon splenic flexure, descending colon, and sigmoid colon. Pathological grade was classified into I/II (well differentiated/moderately differentiated) and III/IV (poorly differentiated/undifferentiated). Histological type was divided into adenocarcinoma and non-adenocarcinoma. The Collaborative Stage Site-Specific Factor (CS-SSF) 4 and CS-SSF 8 were used to extract the information of TDs and PIN, respectively. The TDs were defined as the presence of one or more peritumoral nodules in the pericolorectal adipose tissue of the primary carcinoma without histological evidence of residual lymph nodes in the nodules, which may present as discontinuous diffusion, venous infiltration with extravascular diffusion, or complete lymph node replacement (13). TDs and PIN were classified into existing and non-existing. T stage and N stage were restaged according to the 8^th^ edition American Joint Cancer Committee (AJCC) through CS Extension, CS Lymph Nodes, Regional Nodes Positive and Regional Examined. After restaging, T stage was categorized into T1/T2, T3, T4, and N stage was divided into N0, N1, and N2. The log of positive lymph nodes (LODDS) was calculated by using the following formula: log [(0.5 + the amounts of positive LNs)/(0.5 + the amounts of negative LNs)] (14). The LODDS value in our cohort ranged from −2.30 to 1.95. We used X-tile software to obtain the best cut-off values for LODDS and tumor size. LODDS was grouped into LODDS1 (−2.3 to −0.9), LODDS2 (−0.9 to 0.2), and LODDS3 (0.2–1.95). Tumor size was divided into <5.4 cm, 5.4–6.9 cm, and >6.9 cm. In addition, the selection of variables in the validation group was based on the risk factors involved in the construction of the nomogram.

### Derivation and Internal Validation of the Models

Univariate and multivariate logistic regression analyses were used to predict the risk factors of multiple metastases and derivate models. Variables with P <0.3 in univariate analysis were incorporated into multivariate analysis to build a full model. Then we use stepwise regression to determine the final model. Basing only on the t-statistics of their estimated coefficients, a model was built by continuously adding or deleting variables. This semi-automated process was called stepwise regression, which could provide more powerful information at fingertips and was especially useful for filtering a large number of potential independent variables and/or for fine-tuning the model by storing or removing variables compared with ordinary multiple regression.

The C-index and AUC were used to evaluate the discrimination which was the ability of the predictive model to distinguish populations who have experienced an event from those who have not. On logistic regression model, the value of AUC is the same as that of the C-index. When the AUC is 1, it means the model has a perfect discrimination, while 0.5 represents a random chance of correctly identifying the events. The C-index and its 95% CI were calculated by logistic regression. The degree of calibration is another measure of performance of a prediction model, which tests the degree of agreement between the predicted results and the actual results. The patients were divided into different score groups, and the actual multiple metastasis rate in each group was calculated and was named as observation rate. The predicted rate of each group was calculated according to the mean predicted rate and the standard deviation (SD). The predictive accuracy and discriminative ability of the nomogram were determined by concordance index (C-index) and calibration curve (15). As for the internal verification method, we take the method of bootstrap resampling. Bootstrap resampling is currently one of the most widely used internal verification methods (16, 17). Our nomogram was internally validated by discrimination and calibration with 1,000 times bootstraps. In addition, a new tool to evaluate the value of nomogram in clinical application, called DCA, was used to evaluate the effects of clinical benefits and visualize such effects in the present study (18). The purpose of DCA is to evaluate an individual’s risk of adverse outcomes and to recommend some intervention or treatment for high-risk individuals. Finally, we used AUC and calibration curve to evaluate utility of our nomogram in the validation set.

### Statistical Analysis

R software version 4.0.0 (The R Foundation for Statistical Computing, Vienna, Austria. http://www.r-project.org) was used to run the statistical analysis. The categorical variables were expressed as count (percentage), and chi-square tests were used to compare demographic factors, clinicopathological parameters between the multiple metastases and single metastasis. OS and CSS between the single metastasis and multiple metastases were compared using Kaplan–Meier analysis, and different survival curves were analyzed by log-rank tests. In this study, the R packages including “survival”, “survminer”, “rms”, “MASS”, “pROC”, “Hmisc”, “survivalROC” and “DecisionCurve” were used to draw the Kaplan–Meier curves, build the nomogram, plot the AUC, conduct DCA and calibration curve.

## Result

### Patient Characteristics

In our research, a total of 156,545 cases were obtained from the SEER database. Based on the criteria described above, a total of 5,302 cases were included in the research. The demographic factors and clinicopathological parameters of patients in the present study are summarized in **Table 1**. Briefly, 3,531 patients (66.60%) existed single metastasis, and 1,771 patients (33.40%) had multiple metastases. More than half of the patients were age ≥50 (80.52%), white (74.75%), male (50.87%) and married (56.43%), and LODDS1 (59.20%). The vast majority of patients had positive CEA (77.78%), adenocarcinoma (98.08%), well/moderately differentiated (75.14%) or T3 tumors (58.76%). In contrast, most patients did not have TDs (76.95%), PIN (72.37%). Single metastasis and multiple metastasis groups were different in composition, including sex, marital status, year of diagnosis, tumor site, tumor size, PIN, TDs, histological type, grade, T stage, N stage, and LODDS. Compared with development group, the demographic variables in the validation group were similar, and the clinical variables, including marital status, and race, were significantly different. In the validation group, single metastasis and multiple metastases were different in composition, including tumor site, T stage, LODDS, and grade, which was different from the development group. More detailed information was shown in **Table 1**.

**Table 1:** Clinical and pathological features of metastatic colorectal cancer patients.

Variable	Development Group			Validation Group		
	Entire cohort (N = 5,321)	Single metastases (N = 3,531)	Multiple metastases (N = 1,771)	Entire cohort (N = 120)	Single metastases (N = 80)	Multiple metastases (N = 40)
Age:						
<50	1033 (19.5%)	673 (19.1%)	360 (20.3%)	27 (22.5%)	18 (22.5%)	9 (22.5%)
≥50	4269 (80.5%)	2858 (80.9%)	1411 (79.7%)	93 (77.5%)	62 (77.5%)	31 (77.5%)
Site:						
Right colon	2210 (41.7%)	1374 (38.9%)	836 (47.2%)	50 (41.7%)	21 (26.2%)	29 (72.5%)
Left colon	2490 (47.0%)	1706 (48.3%)	784 (44.3%)	59 (49.2%)	52 (65.0%)	7 (17.5%)
Rectum	602 (11.4%)	451 (12.8%)	151 (8.53%)	11 (9.17%)	7 (8.75%)	4 (10.0%)
CEA:						
Negative	1178 (22.2%)	906 (25.7%)	272 (15.4%)	24 (20.0%)	17 (21.2%)	7 (17.5%)
Positive	4124 (77.8%)	2625 (74.3%)	1499 (84.6%)	96 (80.0%)	63 (78.8%)	33 (82.5%)
T stage:						
T1/T2	207 (3.90%)	174 (4.93%)	33 (1.86%)	4 (3.33%)	4 (5.00%)	0 (0.00%)
T3	3115 (58.8%)	2299 (65.1%)	816 (46.1%)	75 (62.5%)	55 (68.8%)	20 (50.0%)
T4	1980 (37.3%)	1058 (30.0%)	922 (52.1%)	41 (34.2%)	21 (26.2%)	20 (50.0%)
Type:						
Adenocarcinoma	5200 (98.1%)	3491 (98.9%)	1709 (96.5%)	119 (99.2%)	80 (100%)	39 (97.5%)
Non-adenocarcinoma	102 (1.92%)	40 (1.13%)	62 (3.50%)	1 (0.83%)	0 (0.00%)	1 (2.50%)
Tumor deposits:						
No	4080 (77.0%)	2903 (82.2%)	1177 (66.5%)	93 (77.5%)	66 (82.5%)	27 (67.5%)
Yes	1222 (23.0%)	628 (17.8%)	594 (33.5%)	27 (22.5%)	14 (17.5%)	13 (32.5%)
LODDS:						
LODDS1	3139 (59.2%)	2268 (64.2%)	871 (49.2%)	77 (64.2%)	57 (71.2%)	20 (50.0%)
LODDS2	1661 (31.3%)	1022 (28.9%)	639 (36.1%)	31 (25.8%)	20 (25.0%)	11 (27.5%)
LODDS3	502 (9.47%)	241 (6.83%)	261 (14.7%)	12 (10.0%)	3 (3.75%)	9 (22.5%)
Perineural invasion:						
No	3837 (72.4%)	2710 (76.7%)	1127 (63.6%)	92 (76.7%)	63 (78.8%)	29 (72.5%)
Yes	1465 (27.6%)	821 (23.3%)	644 (36.4%)	28 (23.3%)	17 (21.2%)	11 (27.5%)
Grade:						
I/II	3984 (75.1%)	2803 (79.4%)	1181 (66.7%)	96 (80.0%)	73 (91.2%)	23 (57.5%)
III/IV	1318 (24.9%)	728 (20.6%)	590 (33.3%)	24 (20.0%)	7 (8.75%)	17 (42.5%)
Tumor size:						
<5.4	2920 (55.1%)	1969 (55.8%)	951 (53.7%)	67 (55.8%)	45 (56.2%)	22 (55.0%)
5.4–6.9	1242 (23.4%)	866 (24.5%)	376 (21.2%)	21 (17.5%)	16 (20.0%)	5 (12.5%)
>6.9	1140 (21.5%)	696 (19.7%)	444 (25.1%)	32 (26.7%)	19 (23.8%)	13 (32.5%)
Sex:						
Female	2605 (49.1%)	1685 (47.7%)	920 (51.9%)	62 (51.7%)	42 (52.5%)	20 (50.0%)
Male	2697 (50.9%)	1846 (52.3%)	851 (48.1%)	58 (48.3%)	38 (47.5%)	20 (50.0%)
Race:						
White	3963 (74.7%)	2640 (74.8%)	1323 (74.7%)	–	–	–
Black	814 (15.4%)	550 (15.6%)	264 (14.9%)	–	–	–
Other^a^	525 (9.90%)	341 (9.66%)	184 (10.4%)	–	–	–
Insurance:						
Yes	5039 (95.0%)	3363 (95.2%)	1676 (94.6%)	–	–	–
No	263 (4.96%)	168 (4.76%)	95 (5.36%)	–	–	–
Marital status:						
Married	2992 (56.4%)	2035 (57.6%)	957 (54.0%)	103 (85.8%)	68 (85.0%)	35 (87.5%)
Never married	1060 (20.0%)	684 (19.4%)	376 (21.2%)	–	–	–
Other^b^	1250 (23.6%)	812 (23.0%)	438 (24.7%)	17 (14.2%)	12 (15.0%)	5 (12.5%)
N stage:						
N0	1016 (19.2%)	780 (22.1%)	236 (13.3%)	33 (27.5%)	23 (28.7%)	10 (25.0%)
N1	2056 (38.8%)	1424 (40.3%)	632 (35.7%)	46 (38.3%)	34 (42.5%)	12 (30.0%)
N2	2230 (42.1%)	1327 (37.6%)	903 (51.0%)	41 (34.2%)	23 (28.7%)	18 (45.0%)

CEA, carcinoembryonic antigen; LODDS, the log of positive lymph nodes. ^a^Other contains American Indian, Asian, and Pacific Islander. ^b^Other includes divorced, separated, widowed, and unmarried or domestic partner.

### The Impact of Metastasis Status on OS and CSS

**Figure 2:**
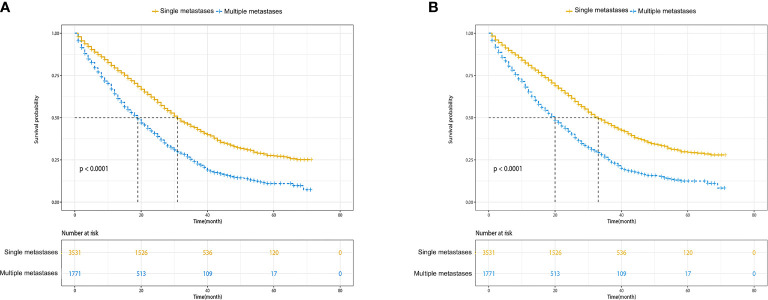
Kaplan–Meier survival curves of survival. **(A)** Overall survival of single metastases *versus* multiple metastases. **(B)** Cancer-specific survival of single metastases *versus* multiple metastases.

The median OS for patients with single metastasis and multiple metastases was 31 (95%CI, 30–33) months and 19 (95%CI, 18–20) months, respectively. The median CSS for patients with single metastasis and multiple metastases was 33 (95%CI, 31–35)and 20 (95%CI, 18–21) months, respectively. The Kaplan–Meier survival curves and log-rank test revealed a significant OS and CSS advantage for single metastasis (p < 0.001 for log-rank test; **Figure 2**). The 1-, 3-, and 5-year OS estimates were 79.4% (95%CI: 78.0–80.9%) and 64.1% (95%CI: 61.7–66.6%), 44.1% (95%CI: 42.0–46.3%) in the single metastasis group and 23.9% (95%CI: 21.3–26.9%), 25.3% (95%CI: 22.8–28.1%) and 10.0%(95%CI: 9.5–14.4%) in the multiple metastases group. The 1-, 3-, and 5-year CSS estimates between single metastasis group and multiple metastases group were 81.0% (95%CI: 79.6–82.4%) and 65.2% (95%CI: 62.8–67.7%), 46.5% (95%CI: 44.3–48.7%) and 25.1% (95%CI: 22.4–28.1%), 29.5% (95%CI: 27.0–32.3%) and 12.0% (95%CI: 9.5–16.2%), respectively.

### Identification of the Risk Factors for Multiple Metastases

**Figure 3:**
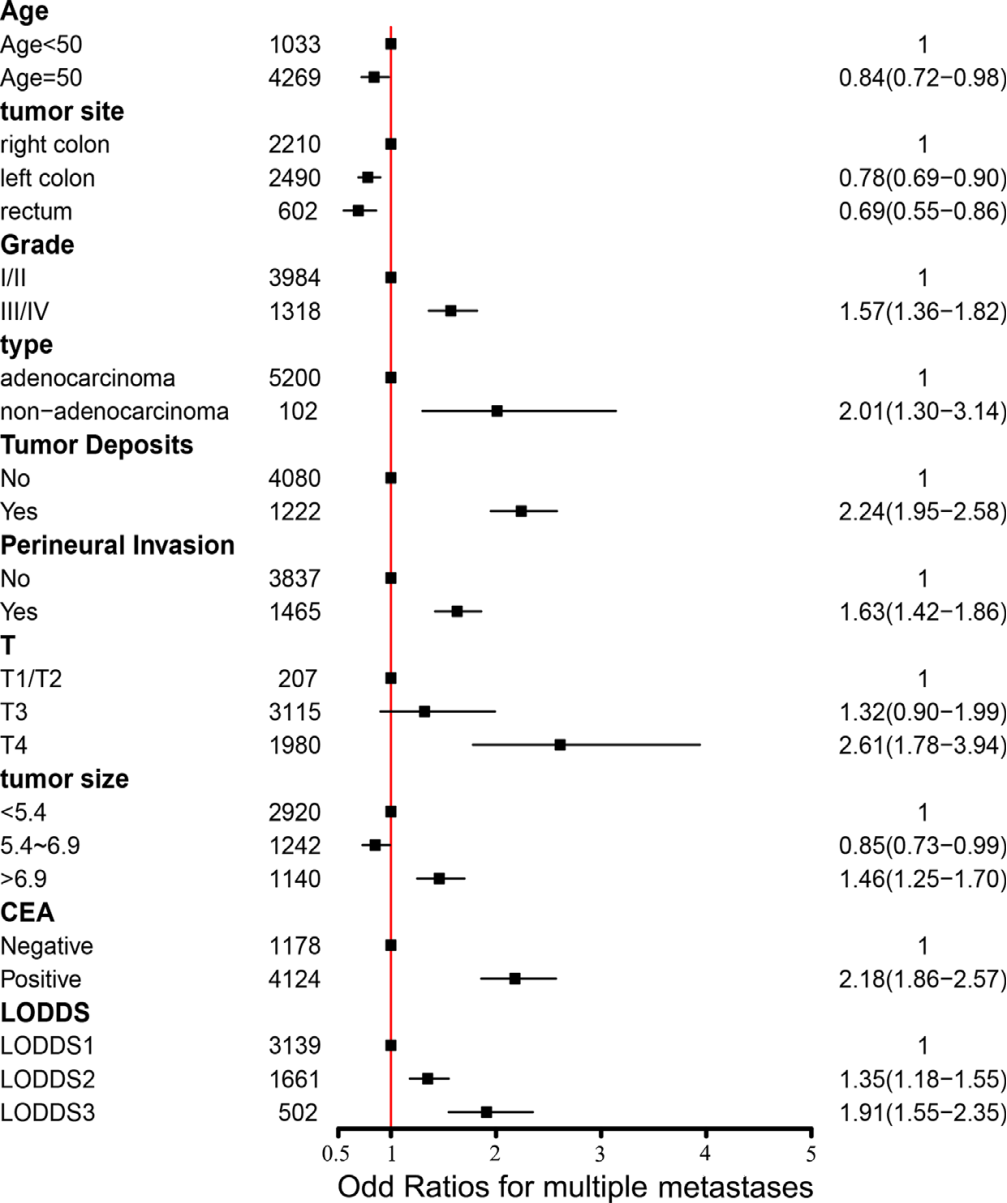
Forest plot with odds ratios for multiple metastases. CEA, carcinoembryonic antigen; LODDS, the log of positive lymph nodes; I/II, well/moderately differentiated; III/IV, poorly/undifferentiated.

We performed logistic regression analysis to explore the risk factors of multiple metastases. Sex, marital status, tumor site, tumor size, serum CEA level, PIN, TDs, grade, histological type, T stage, N stage, and LODDS were statistically significant using univariate logistic regression analysis (**Table 2**). In multivariate stepwise logistic regression analysis, we identified that age, grade, tumor size, PIN, serum CEA level, T stage, TDs, tumor site, histological type, and LODDS were determined as independent risk factors of multiple metastases (**Table 2**). Among them, three indicators including serum positive CEA (OR, 2.18; 95%CI, 1.86–2.57; p < 0.001), T4 stage (OR, 2.61; 95%CI, 1.78–3.94; p < 0.001), non-adenocarcinoma (OR, 2.01; 95%CI, 1.30–3.14; p = 0.002) had the largest impact on multiple metastases (**Figure 3**).

**Table 2:** Univariate/Multivariate Logistic regression analyses of risk factors for multiple metastases in colorectal cancer.

Variable	Variable level	Univariate Logistic regression			Multivariate Stepwise Logistic regression		
		OR	95%CI	p-Value	OR	95%CI	p-Value
Age	<50	Ref			Ref		
	≥50	0.92	0.80–1.07	0.272	0.84	0.72–0.98	0.0305*
Sex	Female	Ref					NI
	Male	0.84	0.75–0.95	0.0037*			
Race	White	Ref					NI
	Black	0.96	0.81–1.12	0.6			
	Other	1.08	0.89–1.30	0.448			
Marital status	Married	Ref					NI
	Never married	1.17	1.01–1.35	0.038*			
	Other	1.15	0.99–1.32	0.0536			
Insurance	Yes	Ref					NI
	No	1.13	0.87–1.47	0.338			
Tumor site	Right colon	Ref			Ref		
	Left colon	0.76	0.67–0.85	<0.001*	0.78	0.69–0.90	<0.001*
	Rectum	0.55	0.45–0.67	<0.001*	0.69	0.55–0.86	<0.001*
Tumor size (cm)	<5.4	Ref			Ref		
	5.4–6.9	0.9	0.78–1.04	0.146	0.85	0.73–0.99	0.033*
	>6.9	1.32	1.15–1.52	<0.001*	1.46	1.25–1.70	<0.001*
CEA	Negative	Ref			Ref		
	Positive	1.9	1.64–2.21	<0.001*	2.18	1.86–2.57	<0.001*
Perineural Invasion	No	Ref			Ref		
	Yes	1.89	1.67–2.14	<0.001*	1.63	1.42–1.86	<0.001*
Tumor Deposits	No	Ref			Ref		
	Yes	2.33	2.05–2.66	<0.001*	2.24	1.95–2.58	<0.001*
Histological type	Adenocarcinoma	Ref			Ref		
	Non-adenocarcinoma	3.17	2.13–4.76	<0.001*	2.01	1.30–3.14	0.002*
Grade	I/II	Ref			Ref		
	III/IV	1.92	1.69–2.19	<0.001*	1.57	1.36–1.82	<0.001*
T	T1/T2	Ref			Ref		
	T3	1.87	1.30–2.78	0.0013	1.32	0.90–1.99	0.16
	T4	4.59	3.18–6.85	<0.001	2.61	1.78–3.94	<0.001*
N	N0	Ref					NI
	N1	1.47	1.24–1.75	<0.001*			
	N2	2.25	1.90–2.67	<0.001*			
LODDS	LODDS1	Ref			Ref		
	LODDS2	1.63	1.44–1.85	<0.001*	1.35	1.18–1.55	<0.001*
	LODDS3	2.82	2.33–3.42	<0.001*	1.91	1.55–2.35	<0.001*

NI, Not included in multivariate stepwise Logistic regression models; OR, odd ratio; CI, confidence interval; CEA, carcinoembryonic antigen; LODDS, the log of positive lymph nodes; I/II, well/moderately differentiated; III/IV, poorly/undifferentiated. *There was a statistical difference.

### Construction of Predictive Nomograms for Multiple Metastases

**Figure 4:**
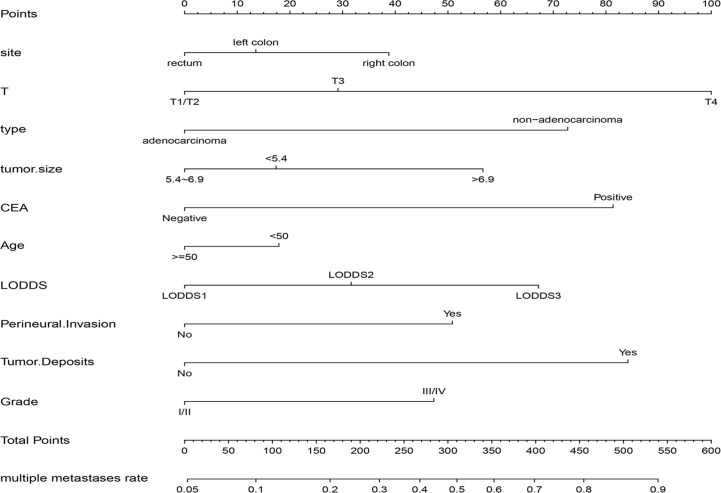
The nomogram for predicting the risk of multiple metastases in CRC. CEA, carcinoembryonic antigen; LODDS, the log of positive lymph nodes.

Based on the multivariable stepwise logistic regression analysis for multiple metastases, all the independent significant risk factors were integrated to build the nomogram for multiple metastases prediction. The predictive nomogram for multiple metastases was illustrated in **Figure 4**.

**Figure 5:**
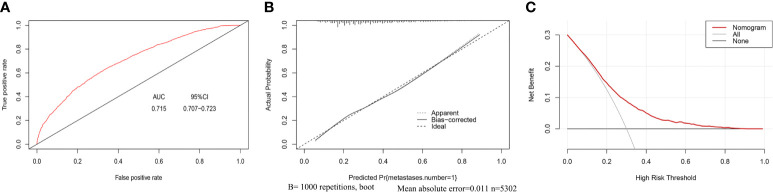
AUC values of ROC predicted multiple metastases **(A)**. The calibration curve of predictive nomograms for predicting multiple metastases **(B)**. Decision curve analysis of the predictive nomogram for predicting multiple metastases **(C)**. (panel **A**) AUC, Area under the curve; 95CI%, 95% confidence interval. (panel **B**) Apparent, Calibration curve of nomogram without bootstrap adjustment; Bias-corrected, Calibration curve of nomogram with 1,000 bootstrap adjustment; Ideal, Ideal calibration curve; (panel **C**) Nomogram: The benefit curve represented by the nomogram; All: Net benefit curve when all samples are multiple metastases; None: Net benefit curve when all samples are single metastasis.

**Figure 6:**
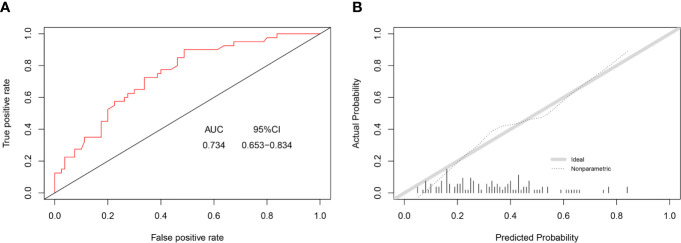
AUC values of ROC predicted multiple metastases **(A)**. The calibration curve of predictive nomograms for predicting multiple metastases **(B)**. (panel **A**) AUC, area under the curve; 95CI%, 95% confidence interval. (panel **B**) Ideal, Ideal calibration curve; Non-parametric, Actual calibration curve of nomogram.

In this study, C-index value and AUC were applied to evaluate the discrimination ability of the nomogram; moreover, C-index value and AUC were adjusted through 1,000 bootstraps as internal validation to ensure that the nomogram had good effect in predicting multiple metastases. The adjusted value of the C-index was 0.715 (95%CI, 0.707–0.723), and as mentioned above, the AUC was the same as the C-index value (**Figure 5A**). Furthermore, the calibration curves of the nomogram for predicting multiple metastases also used 1,000 bootstraps for internal validation. The adjusted calibration curves showed no significant deviation from the reference line, indicating a good level of confidence (**Figure 5B**). As an emerging method for evaluating the prediction of model, DCA suffices the practical needs of clinical decision-making by considering the clinical effects of specific models. The DCA curves for the predictive nomogram are presented in **Figure 5C**. DCA had shown that when the high-risk threshold was 0.3, the application of this nomogram could make nearly 10% of patients get a net benefit without harming other patients, which means that it has good clinical application significance in predicting multiple metastases. Finally, the AUC was 0.734 (95% CI, 0.653–0.834), and calibration curve also showed no significant bias from the reference line in the independent invalidation group, indicating the favorable effects of our nomogram (**Figure 6**).

## Discussion

In this population-based study, we not only identified the risk factors of multiple metastases, but also develop a nomogram to predict multiple metastases in patients with mCRC, which filled the gaps in this field. Our data showed that patients with multiple metastases have a worse prognosis than patients with single metastasis. Further, we identified independent risk factors of multiple metastases, including traditional indicators such as age, grade, tumor size, tumor location, T stage, histological type, and serum CEA level and novel indicators such as PIN, TDs, and LODDS. Subsequently, on the basis of independent risk factors, we established a nomogram for multiple metastasis prediction. The discrimination and calibration of the nomogram were proved, and this nomogram has a good predictive effect on multiple metastases. Moreover, our nomogram has an independent validation group for external verification.

At present, researches are more focused on the effect of specific distant metastatic sites on survival in patients with CRC. Luo et al. found the number of metastatic foci was an independent prognostic factor, and the prognosis of patients with single metastatic foci was better than that of patients with multiple organs involved (19). Although the study also involved the number of metastasis, it focused on specific sites of metastasis, including the liver, lung, bone, and brain and ignored the metastasis of distant lymph nodes and other organs, without strictly distinguishing between single and multiple metastases. Robinson et al. explored the relationship between tumor primary site and metastasis pattern. They found patients with rectal primaries were more inclined to present with synchronous pulmonary metastasis than patients with colon primaries (20). Bingmer et al. explored the association between primary tumor location and overall survival in CRC of liver metastases and found that right colon cancer had significantly worse survival than left colon cancer (21). However, the above-mentioned studies mainly focus on specific single or two metastasis sites, such as liver metastasis and pulmonary metastasis. There is a lack of an overall research of multiple metastases, especially for the risk factors of multiple metastases, even though previous studies have identified multiple metastases as an independent prognostic factor.

Based on multiple metastases as independent prognostic factors, we further explored the independent risk factors of multiple metastases in mCRC, which was ignored in previous studies. We totally identified ten independent risk factors of multiple metastases, including age, grade, tumor size, tumor site, PNI, serum CEA level, histological type, T stage, TDs, and LODDS. Among them, three indicators including positive serum CEA level (HR, 2.18; 95%CI, 1.86–2.57; *p* < 0.001), T4 stage (HR, 2.61; 95%CI, 1.78–3.94; *p* < 0.001), existing TDs (HR, 2.24; 95%CI, 1.95–2.58; *p* < 0.001) had the largest impact on the multiple metastases (**Figure 3**).

Based on these independent risk factors, we firstly proposed a nomogram to predict multiple metastases. Compared with the other nomograms involving distant metastasis of CRC, our nomogram focused on the prediction of multiple metastases, often neglected in past research. More importantly, our nomogram not only introduced common clinicopathological parameters but also included some novel indicators, including LODDS, TDs and PIN, which were not available in previous relevant nomograms. More details about nomograms^’^ differences were summarized in **Table S2**.

LODDS is an emerging indicator that has been evaluated by many scholars as lymph node stage and may take on a better role than the AJCC lymph node staging system version (14). Zhang et al. proved that the new LODDS classification was an independent prognostic factor for CRC patients, which required the calculation of additional risk group stratification through internal and external databases (22). LODDS was identified as an independent risk factor for multiple metastases in our study, which was not involved in previous studies. At present, more and more study focused on tumor deposits. Lord et al. explored the significance of TDs in neoadjuvant therapy for rectal cancer. They found that, similar to untreated patients, the presence of TDS in rectal cancer patients after neoadjuvant therapy was related to disease progression and poor prognosis (23). Pricolo et al. revealed the TDs and PIN were associated with worse survival in stage III colon cancer, and they recommended a combination of TDs and lymph node examination as “N2c” (24). D’Souza et al. found that the presence of tumor deposits on CT was associated with disease recurrence and had the strongest association with poor outcome in sigmoid colon cancer (25). Lino-Silva et al. found tumor deposits were invariably associated with worse prognosis, especially with the increasing rate of distant metastasis (26). Our research found TD was an independent risk factor of multiple metastases in mCRC, which extends the research direction of TDs. Furthermore, the study has found that serum CEA level is a prognostic factor and an ideal biomarker for CRC patients (27). Routine CEA monitoring was made use in postoperative follow-up to monitor recurrence and distant metastasis after CRC resection surgery. As multivariate analysis manifested, mCRC patients with positive serum CEA levels were more inclined to have higher multiple metastatic probabilities. In related studies, histological differentiation had been defined as an important feature in evaluating the advantages of adjuvant chemotherapy (28). The results of this study indicate that when the grade of the tumor shows poorly differentiated/undifferentiated grade, it is more likely to cause multiple metastases. Some scholars suggested that patients with higher T stage suffered from a higher risk of liver metastases (29). The higher T stage was related to deeper infiltration, which might lead to the metastasis of malignant tumor cells to the blood vessels. Multivariate research has revealed that higher T stage was associated with a higher risk of multiple metastases. In parallel results, tumor size was an independent factor for OS in patients with ulcerative infiltrating colorectal adenocarcinoma (30). Previous studies have shown that younger patients were apt to experience a higher risk of lung, liver, and bone metastases (19, 29). Our research extends this conclusion to multiple metastases. In addition, the rates of pulmonary and liver metastasis in left CRC patients were significantly higher than those in right CRC patient, but the prognosis was better than those with right CRC in terms of OS, which was slightly different from our research (29). In our study, right CRC had a highest rate of multiple metastases. In short, our study extended these variables’ application to the risk factors of multiple metastases. Regarding age, we need to emphasize that because there are only 246 patients older than 85 years old, and there was no significant difference in the number of metastases from patients aged 50 to 85 years old, so we included them as a whole into the population older than 50 years old. However, it must be noted that elderly patients, especially those over 85 years old, usually had a poorer basic state of the whole body and were often accompanied by other diseases. This required further detailed research and could also help improve the quality of the model. In addition, previous studies have found that racial differences affect the metastasis of specific sites. Compared with blacks and whites, other races have a relatively lower risk of metastasis (9, 12). However, in our research, we did not find the impact of racial differences on the risk of multiple metastases. Therefore, race is not included in our model, but objectively there is a huge difference in race between the development group and the validation group. Limited to the number of patients of other races, especially the number of Asians especially (only 56 cases) in SEER database, the influence of race on multiple metastases had not been fully explored. Obviously, this would be one of the directions of future research, which could help to further optimize the quality of the model.

## Limitations

We acknowledge that this study had some limitations. First, as a large retrospective study, these findings must take into account the inherent selection bias. The data provided by the SEER database allow researchers to explore associations that are difficult to uncover because of the limited sample size. However, the unobservable confounders limited the interpretation of observational data, even though we tried to reduce the bias with multivariable analysis. Second, there are some conflicting data about metastases information in the SEER program. For example, a patient has both liver and lung metastases, but the patient M stage showed “M1a”, which may affect data collation and result analysis. Third, the number of accessible variables provided by the SEER database was limited. For instance, salvage therapies, clinical treatment response, specific chemotherapeutic agents, and immunotherapy were not included in the data, which may otherwise affect the interpretation of results. In addition, the SEER program lacks several important biomarker expression states, such as MSI, NRAS, KRAS, and BRAF, which were closely associated with metastases in colorectal cancer. Comparing with other nomogram relating to metastasis, the AUC of our nomogram was relatively low, prompting us to further optimize the model parameters. Finally, although we conducted external verification, due to the limitation of the number of cases in the external verification group, there may be unknown deviations.

Despite these limitations, our study further confirmed by stratified analysis that the degree of metastasis was a prognostic factor in mCRC. Patients with multiple metastases had poorer OS and CSS than those with simple metastasis. Furthermore, we investigated independent risk factors for multiple metastases in mCRC and constructed a nomogram based on these independent risk factors to predict multiple metastases, which had been ignored in previous studies. In general, our study focused on the risk factors for multiple metastases and the construction of a nomogram, which filled in the gaps in previous similar studies.

## Data Availability Statement

The datasets presented in this study can be found in online repositories. The names of the repository/repositories and accession number(s) can be found in the article/**Supplementary Material**. The validation set data is available from the corresponding author.

## Ethics Statement

The study was approved by the Ethics Committee of the Renmin hospital of Wuhan University, and the requirement of written informed consent was waived.

## Author Contributions

TF supervised the project. YHG and TF conceived and designed the original study. YHG and RSX downloaded and analyzed the data of the training set. JR collected data from the external validation set and participated in part of the data analysis and final revision of the manuscript. YHG, RSX and JR contributed to the interpretation of the data. JR and WS were the main drafters of the manuscript, with the participation of YHG and RSX. TF, YHG, JR and WL revised the manuscript. All authors contributed to the article and approved the submitted version.

## Conflict of Interest

The authors declare that the research was conducted in the absence of any commercial or financial relationships that could be construed as a potential conflict of interest.

## Supplementary Material

Supplementary Table 1 Exclusion criteria and reasons that we excluded patients. Abbreviations: CEA, carcinoembryonic antigen; LODDS, the log of positive lymph nodes; AJCC, American Joint Cancer Committee.

Supplementary Table 2 Comparison of nomograms related to predicting colorectal cancer metastasis by using SEER. Abbreviations: CEA, Carcinoembryonic antigen; LODDS, the log of positive lymph nodes; TDs, Tumor Deposits; PI, Perineural Invasion; LNH, lymph nodes harvested; AUC, Area under the curve.
