# Supplementary material for: A Nomogram for Predicting Multiple Metastases in Metastatic Colorectal Cancer Patients: A Large Population-Based Study
Source: Front Oncol. 2021 May 13;11:633995. doi: 10.3389/fonc.2021.633995 (PMC8155489; doi:10.3389/fonc.2021.633995)
Supplement: Supplementary Table 1 — Exclusion criteria and reasons that we excluded patients. Abbreviations: CEA, carcinoembryonic antigen; LODDS, the log of positive lymph nodes; AJCC, American Joint Cancer Committee. [file Table_1.docx]

| Table S1. Exclusion criteria and reasons that we excluded patients. | |
| --- | --- |
| Exclusion criteria | Reasons |
| Patients for whom colorectal cancer was not the first malignant tumor. | Inclusion of patients with metachronous colorectal cancer could complicate the results; for example, metastatic sites might be influenced by antecedent tumors. |
| Patients who were less than 18 years old. | Childhood colorectal cancer is rare and young patients are different from adult patients in terms of treatments, prognosis and outcomes. |
| Patients for whom information about distant metastases was unavailable. | The information of distant metastases was the focus of our study. |
| Patients who did not have histologically confirmed stage IV colorectal cancer. | Patients with distant metastases were defined as having stage IV colorectal cancer based on the AJCC staging system in the SEER program. |
| Patients whose information about age, marital status, race and sex, insurance, tumor location, tumor size, serum CEA level, perineural invasion, tumor deposits, histological type, grade, T stage, N stage, regional node examination information, survival time was unavailable. | Survival time was required to plot Kaplan-Meier curves, regional node examination information needed calculate LODDS, and the other variables were those we wanted to adjust in the univariate and multivariate logistic model employed in our study. |

Abbreviations: CEA, carcinoembryonic antigen; LODDS, the log of positive lymph nodes; AJCC, American Joint Cancer Committee
