# Supplementary material for: A Nomogram for Predicting Multiple Metastases in Metastatic Colorectal Cancer Patients: A Large Population-Based Study
Source: Front Oncol. 2021 May 13;11:633995. doi: 10.3389/fonc.2021.633995 (PMC8155489; doi:10.3389/fonc.2021.633995)
Supplement: Supplementary Table 2 — Comparison of nomograms related to predicting colorectal cancer metastasis by using SEER. Abbreviations: CEA, Carcinoembryonic antigen; LODDS, the log of positive lymph nodes; TDs, Tumor Deposits; PI, Perineural Invasion; LNH, lymph nodes harvested; AUC, Area under the curve. [file Table_2.docx]

| **Table 2S. Comparison of nomograms related to predicting colorectal cancer metastasis by using SEER** | | | |
| --- | --- | --- | --- |
| **Model** | Nomogram of Luo el (12) | Nomogram of Mo el (9) | Our nomogram |
| **Purpose** | Predicting specific site metastasis, such as liver, lung, bone, brain | Predicting specific site metastasis, such as liver, lung, bone, brain | Predicting multiple metastases |
| **Risk factors for building nomogram** | Age, Sex, Race, Insurance, Site, Grade, T, N, CEA, Specific metastatic site | Gender, Site, Grade, Age, Histological type, Tumor size, CEA, T, N, LNH | Site, T, Histological type, Tumor size, CEA, Age, LODDS, Perineural invasion, Tumor deposits, Grade |
| **Model AUC (%)** | Liver: 86.7; Lung: 89.2; Bone: 91.1; Brain: 88.3 | Liver: 82.5; Lung: 79.8; Bone:82.3; Brain:78.6 | 71.5 |
| **Strengths** | 1.Predicting specific site metastasis 2.Modeling with a large sample 3.AUC shows a better prediction effect 4.Optimizing the previous models | 1.Predicting specific site metastasis 2.Modeling with a large sample 3.AUC shows a better prediction effect | 1.Predicting multiple metastases, and filling the gap in this field 2.Modeling with a large sample  3.A certain amount of external validation set |
| **Limitations** | 1. Unable to predict multiple metastases; 2.No external validation set | 1. Unable to predict multiple metastases 2.No external validation set | 1.Uable to predict specific site metastasis 2.Needing optimize model to improve AUC |

Abbreviations: CEA: Carcinoembryonic antigen; LODDS: the log of positive lymph nodes; TDs: Tumor Deposits; PI: Perineural Invasion; LNH: lymph nodes harvested; AUC: Area under the curve
